# Immediate Emergency Department Diagnosis of Pyloric Stenosis with Point-of-care Ultrasound

**DOI:** 10.5811/cpcem.2017.9.35016

**Published:** 2017-11-03

**Authors:** Nicole Dorinzi, Justine Pagenhardt, Melinda Sharon, Kristine Robinson, Erin Setzer, Nicolas Denne, Joseph Minardi

**Affiliations:** West Virginia University, Department of Emergency Medicine, Morgantown, West Virginia

## Abstract

A 15-day-old male who was born at term presented with non-bilious projectile vomiting. He was nontoxic and his abdomen was benign without masses. Point-of-care ultrasound (POCUS) showed hypertrophic pyloric stenosis (HPS). Typical findings include target sign; pyloric muscle thickness greater than three millimeters (mm); channel length greater than 15–18 mm; and lack of gastric emptying. The patient was admitted; consultative ultrasound (US) was negative, but repeated 48 hours later for persistent vomiting. This second US was interpreted as HPS, which was confirmed surgically. Pyloromyotomy was successful. Few reports describe POCUS by general emergency physicians to diagnose HPS. Here, we emphasize the value in repeat US for patients with persistent symptoms.

## INTRODUCTION

Neonatal vomiting is common in the emergency department (ED), and may represent a life-threatening condition. Hypertrophic pyloric stenosis (HPS) is among the important differential considerations. Ultrasound (US) is recommended as the initial imaging modality for HPS. Consultative US from radiology is frequently not available after hours or on weekends. Point-of-care ultrasound (POCUS) allows treating clinicians the ability to make this diagnosis without delay, potentially improving patient care, decreasing the need for other evaluations, and expediting therapy.

The technique and sonographic findings are relatively simple, and can be accurately identified by physicians even with limited US experience. A single-center study of trained pediatric emergency physicians (EP) demonstrated the ability to accurately make the diagnosis at the bedside.^1^ Otherwise, only a single case series by general EPs exists describing the findings.^2^ Our case gives further evidence to the ability of general EPs in making this diagnosis using POCUS, and emphasizes the dynamic nature of the findings and utility of serial US in certain cases.

## CASE REPORT

A 15-day-old male who was born at term presented to the ED from home with parents for complaint of vomiting. For five days, he had been experiencing projectile vomiting that was non-bilious and non-bloody. His mother denied stool changes, recent illnesses, or fevers. The patient’s birth and past medical history were unremarkable. He was a first-born son by spontaneous term vaginal delivery. The patient’s immunizations were up to date.

**Image 1:**
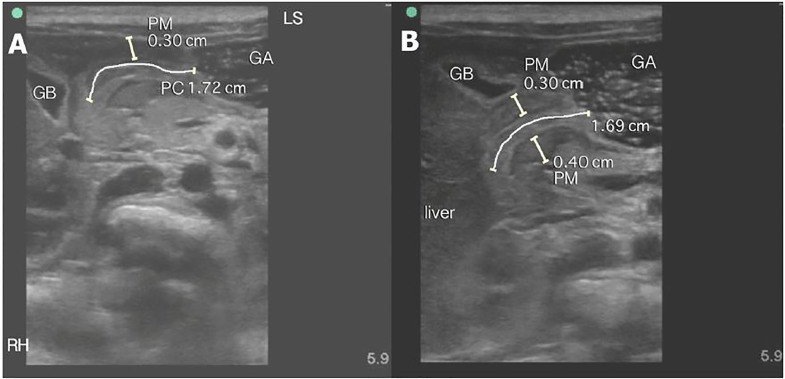
Pyloric stenosis long axis These are long-axis views of the pylorus showing a thickened, elongated pyloric channel (PC) (A), and (B) with measurements of channel length greater than 17 mm and pyloric muscle (PM) thickness greater than three mm. GA – gastric antrum, GB - gallbladder. Orientation is oblique. LS represents the relative position of the patient’s left shoulder and RH represents the relative position of the patient’s right hip.

**Image 2:**
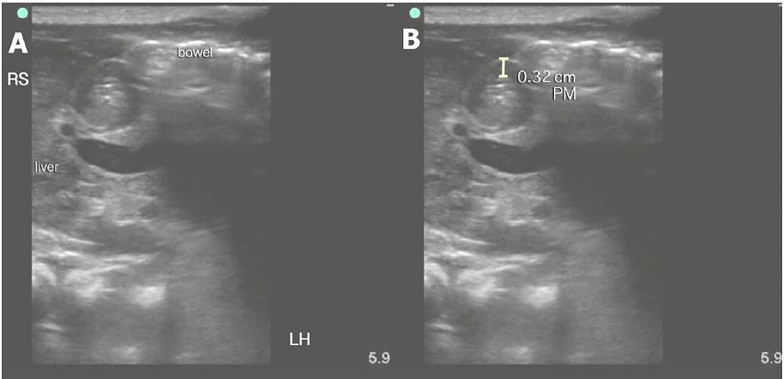
Pyloric stenosis short axis These are short-axis views of the pylorus with findings of a thickened pyloric muscle (PM) (A). (B) A measurement of the pylorus muscle (PM) wall greater than three mm is shown here. Orientation is oblique. RS represents the relative position of the patient’s right shoulder and LH represents the relative position of the patient’s left hip.

On examination, the patient was nontoxic, abdomen had normal bowel sounds, no tenderness, or palpable masses. The remainder of his examination including genitourinary exam was unremarkable. Laboratory results were also unremarkable. POCUS was performed, revealing a thickened, elongated pyloric muscle, and absent gastric emptying with feeding (Images 1 and 2), suggesting the diagnosis of HPS. The patient was treated supportively with intravenous fluids and admitted; he underwent a consultative radiology US the next morning. This study was interpreted as negative. When the patient failed to tolerate oral feeds, a repeat consultative US was performed 48 hours later, which was interpreted as positive for HPS. He was taken to the operating room, where pyloric stenosis was “clearly present” per the operative note. Pyloromyotomy was performed, and the patient recovered well.

## DISCUSSION

HPS is a surgical emergency, and is the most common cause of intestinal obstruction in infants.^3^,^4^ For unknown reasons the pylorus hypertrophies after birth and causes progressive gastric outlet obstruction. The incidence is approximately 2–5 per 1,000 live births; the risk is four times higher in boys than girls.^3^,^4^ Infants most commonly present between two and six weeks of age.^3^ HPS should be considered in any infant less than six months of age with vomiting. Classically, vomiting is described as progressive, non-bilious and projectile in nature. It may lead to poor feeding, weight loss and dehydration. Traditionally, HPS was diagnosed with palpation of an olive-sized mass in the right upper quadrant of the infant with vomiting. However, this physical exam finding is detected variably in 11–51% of cases.^3^ Additionally, the classic laboratory findings of hypokalemia and hypochloremic metabolic alkalosis are only seen in 9–36% of patients.^4^

In a prospective study pediatric EPs using POCUS after limited training were able to diagnose HPS with sensitivity and specificity approaching 100%, or as accurately as radiologists.^1^ Another case series has documented the ability of general EPs to diagnose HPS using POCUS.^2^ In addition, surgeons using POCUS have demonstrated the ability to obtain accurate pyloric measurements, and make this diagnosis at the bedside.^5^

To perform POCUS of the pylorus, the infant should be placed in the caregiver’s lap in the right lateral decubitus position. Warmed US gel should be used to decrease discomfort, and the infant should be fed an electrolyte solution. Using a high-frequency linear transducer, obtain transverse images through the epigastrium, identifying the liver and gallbladder to the right and stomach to the left as useful landmarks. As the stomach becomes distended, it should be followed medially to the gastric antrum, which will lead to the muscular pylorus. Once identified, the transducer should be rotated until it can be visualized in its longest axis, and the pyloric channel length should be measured.

Measurements should include the thickness of the hypoechoic muscular layer, which is external to and should not include the hyperechoic pyloric channel. Measurements of the muscular layer thickness can and should be obtained in the longitudinal and transverse planes. The pylorus should be observed for the passage of gastric contents where the hypoechoic fluid from the stomach will be observed moving through the pyloric channel. Some echogenic air bubbles may also be observed.

**CPC-EM Capsule**

What do we already know about this clinical entity?

Data is limited on point-of-care ultrasound (POCUS) by emergency physicians (EP) to diagnose hypertrophic pyloric stenosis (HPS).

What makes this presentation of disease reportable?

Only one series has reported general EPs using POCUS to diagnose HPS. Review of the concept, technique, and findings should assist others in adding this skill.

What is the major learning point?

General EPs using POCUS can diagnose HPS. Technique: linear probe, right lower decubitus position, during feeding. Findings: elongated, thickened pylorus; failure to pass gastric contents.

How might this improve emergency medicine practice?

EPs incorporating POCUS for HPS into their practice should allow a more rapid diagnosis, earlier therapy, and more efficient use of resources.

Normal sonographic findings include a pylorus muscle thickness of less than two mm with channel length less than 15 mm. Pathologic findings are an increased overall diameter, thickened muscular walls (greater than three mm), an elongated pylorus (greater than 17 mm), and lack of gastric emptying. Other findings include the “shoulder sign” and “antral nipple.” The “shoulder sign” is the protrusion of the rounded hypertrophied pyloric muscle into the gastric antrum giving the appearance of a shoulder. The “antral nipple” sign is protrusion of the thinner pyloric channel mucosa into the gastric antrum. 2 In some cases, pylorospasm may mimic the sonographic findings of HPS, so additional images may be obtained after 15–20 minutes as part of the initial exam if measurements are equivocal. As in this case, where a later consultative study was interpreted as negative, repeat imaging should always be performed when clinical symptoms are persistent. Initial management of HPS is supportive with fluid and electrolyte replacement. Surgical pyloromyotomy is the definitive treatment.

## CONCLUSION

We present a case of hypertrophic pyloric stenosis diagnosed immediately at the bedside by an EP using point-of-care ultrasound. HPS is an important diagnosis for the EP to consider in at-risk patients. The sonographic findings are easily obtained and interpreted by EPs, even with limited training. Earlier diagnosis or exclusion of this disease process should lead to more focused patient evaluations, consultation and management while decreasing the use of other resources.
